# Flexible SAW Microfluidic Devices as Wearable pH Sensors Based on ZnO Nanoparticles

**DOI:** 10.3390/nano11061479

**Published:** 2021-06-03

**Authors:** Luigi Piro, Leonardo Lamanna, Francesco Guido, Antonio Balena, Massimo Mariello, Francesco Rizzi, Massimo De Vittorio

**Affiliations:** 1 Center for Biomolecular Nanotechnologies, Istituto Italiano di Tecnologia, 73010 Arnesano, Italy; francesco.guido@iit.it (F.G.); antonio.balena@iit.it (A.B.); massimo.mariello@iit.it (M.M.); francesco.rizzi@iit.it (F.R.); massimo.devittorio@iit.it (M.D.V.); 2 Department of Innovation Engineering, Campus Ecotekne, University of Salento, 73100 Lecce, Italy; 3 Center for Nano Science and Technology, Istituto Italiano di Tecnologia, 20133 Milan, Italy; leonardo.lamanna@iit.it

**Keywords:** SAW, wearable device, flexible, biosensor, PEN, pH ZnO nanoparticles

## Abstract

In this work, a new flexible and biocompatible microfluidic pH sensor based on surface acoustic waves (SAWs) is presented. The device consists of polyethylene naphthalate (PEN) as a flexible substrate on which aluminum nitride (AlN) has been deposited as a piezoelectric material. The fabrication of suitable interdigitated transducers (IDTs) generates Lamb waves (L-SAW) with a center frequency ≈500 MHz traveling in the active region. A SU-8 microfluidics employing ZnO nanoparticles (NPs) functionalization as a pH-sensitive layer is fabricated between the IDTs, causing a shift in the L-SAW resonance frequency as a function of the change in pH values. The obtained sensitivity of ≈30 kHz/pH from pH 7 to pH 2 demonstrates the high potential of flexible SAW devices to be used in the measurement of pH in fluids and biosensing.

## 1. Introduction

The development of biocompatible and flexible micro-electro-mechanical system (MEMS) sensors has increased over the last decade [1]. Surface acoustic wave (SAW)-based devices represent one of the main building blocks in MEMS and have applications in many fields, mostly the development of biosensors for many different purposes (pH, heavy metals concentrations) [2,3], and different physical (light intensity, temperature, deformation) [4,5,6,7] and biological (proteins, small molecule or cell concentrations) [8,9,10] parameters. Usually, SAW devices are fabricated on thick piezoelectric crystals as LiNbO_3_ or LiTaO_3_ [11]. These materials show excellent piezoelectric properties, but their high stiffness and expensive cost make them unsuitable for wearable applications. Recent progress in thin piezoelectric film deposition techniques has allowed thin AlN or ZnO to be deposited on film on soft substrates [1]. The demand for flexible and skin-compliant materials has increased in the last decade, and new flexible materials such as Kapton [12] or polyethylene naphthalate (PEN) [13] have been exploited. The introduction of such low-cost and flexible materials and the remote-control capability of SAW-based devices have made them good candidates for biosensors applications. 

pH measurement is fundamental in many fields such as agriculture, chemistry, pharmaceuticals and biochemical processes, and, above all, its value is one of the most important parameters for monitoring physiological, biological and the medical state of human health [14]. Human fluids (sweat, saliva, blood, etc.) have their own physiological pH value range. If the pH values are detected out of the physiological range, this could be an efficient way to alert for illnesses and dysfunctions. For these reasons, many different pH sensors have been developed, based especially on electrochemical (potentiometric and conductometric) [15] and non-electrochemical (optical and calorimetric) [16] principles. Here, an innovative pH sensor based on SAWs is shown. 

Polyethylene naphthalate (PEN), a thermoplastic polyester widely used in electronics, packaging, textile and industrial fibers, has been used as a substrate for SAW fabrication. PEN is a flexible, transparent and biocompatible material with good mechanical and electrical insulation properties [17]. It is also resistant to heat, moisture and many chemical solvents used in microfabrication processes [18]. Recently, aluminum nitride (AlN) has been used as a piezoelectric material in combination with PEN, which allows the generation and propagation of SAWs on the surface of the thin piezoelectric layer [19]. AlN has good piezoelectric and dielectric properties [20] and it is biocompatible [21]. In particular, c-axis (0002)-orientated AlN allows two principal wave propagation modes: Rayleigh waves (R-SAWs) and Lamb waves (L-SAWs). The R-SAWs are favored in the AlN that is grown on rigid substrates (usually silicon) and propagate at a phase velocity of about 5000 m/s, whereas the L-SAWs are favored in membranes and thin films and, when grown on flexible substrates, travel at a phase velocity of about 10000 m/s [1]. Therefore, AlN presents a high SAW propagation velocity and a high breakdown voltage. There are several deposition techniques used to deposit AlN, such as laser ablation, chemical vapor deposition and reactive sputtering [22,23]. Thanks to recent progress, the last technique allows the deposition of good c-axis (0002)-orientated crystals of AlN at a low temperature [13]. These features make PEN/AlN a great combination for SAW devices. This new platform has been applied as an acoustic-based sensor for temperature, strain, light and E. coli cells’ quantification [4,5,24]. The fundamental challenge is to integrate a microfluidic channel directly onto a SAW device in order to obtain a sensor that detects in real-time. The introduction of a microfluidic system on a SAW-based device and the functionalization of the microchannel active region allows the detection of many bio-chemical parameters in a liquid solution through SAW resonance frequency shift. The main issue in SAW-based lab-on-chip microfluidic devices is that when a liquid sample is in contact with IDTs it dampens the signal [25]. Hence, avoiding direct contact between the fluid sample and the transducers by introducing microfluidics, it has been possible to measure the S21 signal and its variation depending on the chemical or biological variations of the samples. A ≈300 μm thick microchannel has been fabricated directly on a SAW device and then functionalized with ZnO nanoparticles (NPs), which have become the most used metal oxide nanoparticles for biological applications due to their good biocompatibility, low toxicity and low cost. ZnO NPs have applicability in many fields including the rubber industry, the pharmaceutical and cosmetic industries, and the textile and electronic (photoelectronic, UV laser, solar cells, etc.) industries [26]. In this work, they are used as a pH-sensitive layer due to their ability to change their conductivity depending on the pH value (in the acid region).

The working principle of an SAW device is based on the application of a radiofrequency (RF) signal to the metallic IDTs, which allows the generation and propagation of SAWs along the piezoelectric surface. The center frequency of these devices is given by Equation (1):

(1) $$f_{0}=v/\lambda$$

where the velocity v depends on the substrate’s nature and the type of the traveling wave, while the wavelength λ depends on the period of the IDT.

The fundamental biosensing principle using acoustic waves is the measurement of the changes in the propagation velocity of the waves through the changes in the resonant frequency or phase angle. These variations can be due to extrinsic factors such as temperature, UV or IR light intensity, pH, mass loading, pressure, and many others. Equation (2) summarizes the factors that can contribute to the acoustic wave velocity shift:

(2) $$\frac{\Delta f}{\mathrm{fo}}=\frac{\Delta v}{\mathrm{vacoustic}}=\frac{1}{v}\left(\frac{\mathrm{dv}}{\mathrm{dm}}\Delta m+\frac{\mathrm{dv}}{d\sigma }\;\Delta \sigma +\frac{\mathrm{dv}}{\mathrm{dc}}\;\Delta c+\frac{\mathrm{dv}}{d\varepsilon }\;\Delta \varepsilon +\frac{\mathrm{dv}}{\mathrm{dT}}\;\Delta T+\frac{\mathrm{dv}}{\mathrm{dP}}\;\Delta P+\frac{\mathrm{dv}}{d\eta }\Delta \eta +\frac{\mathrm{dv}}{d\rho }\;\Delta \rho \;+\ldots \right)$$

where Δm is the change in mass load, Δσ is the change in conductivity, Δc is the change in mechanical constant, Δε is the change in dielectric constant, ΔT is the change in temperature, ΔP is the change in pressure, Δη is the change in viscosity and Δρ is the change in density.

To detect a change in the H_3_O^+^ in a solution, a pH-sensitive layer is necessary. In this work ZnO NPs have been used for this role. The significant difference in electronegativity between zinc (1.65) and oxygen (3.44) gives a sufficient negative charge on the latter to strip a proton from a neighboring H_3_O^+^ [27] following Equation (3):

(3) $${\mathrm{ZnO}}_{\left(s\right)}+H_{3}O^{+}\;\overset{\mathrm{Ka}}{⇄}\;{\mathrm{ZnOH}}^{+}+H_{2}O$$

where Ka is the dissociation constant of the equation.

The oxygen present in the atmosphere is absorbed onto the surface of the ZnO NPs layer as negatively charged ions capturing free electrons from the film. With the proton-stripping reaction, surface oxygen ions are neutralized and the conductivity, σ, increases.

In the case of a layered structure Equation (2) becomes Equation (4):

(4) $$\frac{\Delta v}{\mathrm{vacoustic}}=-c_{m}f_{0}{\Delta \rho }_{s}+{\;c}_{e}f_{0}h\Delta \left[\frac{\left(\frac{4\mu }{v_{0}^{2}}\right)\left(\eta +\mu \right)}{\eta +2\mu \;}\right]-\left({\frac{K}{2}}^{2}\right)\Delta \left(\frac{\sigma^{2}}{\sigma^{2}+v_{0}^{2}C_{s}^{2}}\right)$$

where *f*_0_ is the center frequency of the sensor, cm is the sensitive coefficient for the mass, $\rho_{s}$ is the mass per unit area, ce is the sensitive coefficient for elasticity, μ and η are the shear and bulk moduli of the film, σ is the conductivity of ZnO NPs, and K² and Cs the electromechanical coupling coefficient and the capacitance per length of the substrate material, respectively. All these terms contribute to the frequency shift.

Given that the mass and electric constant do not change during the interaction between the NPs and the solution, the variation in SAW velocity is influenced by a change in conductivity of the ZnO NPs layer, and Equation (4) assumes the form of Equation (5).

(5) $$\frac{\Delta v}{\mathrm{vacoustic}}=-\left({\frac{K}{2}}^{2}\right)\Delta \left(\frac{\sigma^{2}}{\sigma^{2}+v_{0}^{2}C_{s}^{2}}\right)$$

Hence, a conductive layer has been formed in the microchannel.

ZnO NPs are deposited inside a ≈300 μm thick and 250 μm large microchannel, exactly aligned between the two IDTs to avoid direct contact between the IDT and the liquid solution and the consequent damping of the wave. This variation in the wave velocity changes the resonance frequency of SAWs.

In this paper a novel hybrid PEN/AlN/SU-8 microfluidic sensor is presented. The fabrication and the characterization of a microfluidic channel directly on AlN/PEN flexible SAW devices are described. Through chemical–physical tests, the microchannel’s wettability and, using mechanical tests, its conformability for wearable applications have been demonstrated. In particular, the microfluidic channel has been placed between the two interdigital transducers (IDTs) to let the analyzed fluids cross the pH-sensitive ZnO-NPs functionalized SAW-active region. The high-sensitivity of L-SAW on polymeric substrate for pH detection has been demonstrated.

## 2. Materials and Methods

### 2.1. Sample Preparation

AlN-based SAW devices were fabricated on 125 μm thick polyethylene naphtalate (PEN), purchased from Teonex (code Q65HA) (Wilmington, DE, USA) with one pretreated surface, which improves the growth adhesion for material deposition. The PEN samples (2.5 × 2.5 cm^2^) were fixed on silicon substrate, by a removable PDMS layer, in order to allow a uniform deposition and reduce the temperature stress that occurred during AlN multistep deposition. Hence, the overall 4.5 μm thick AlN layer was grown on PEN samples through magnetron sputtering deposition (LAB18, Kurt J. Lesker Company, Jefferson Hills, PA, USA), achieving a film of 4.5 μm thickness and a good effective piezoelectric coefficient (d33) ranging between 2.12 and 3.29 pm/V [13]. Samples were then washed in acetone, isopropanol and water, respectively, and then dried with N_2_ flow.

### 2.2. Lithography Processes for Microfabrication of PEN/AlN/SU-8-Based Device

In order to obtain a Lamb wave at ≈500 MHz, each PEN/AlN SAW device has been designed with two identical IDTs with the parameters reported in Table 1.

**Figure 1:**
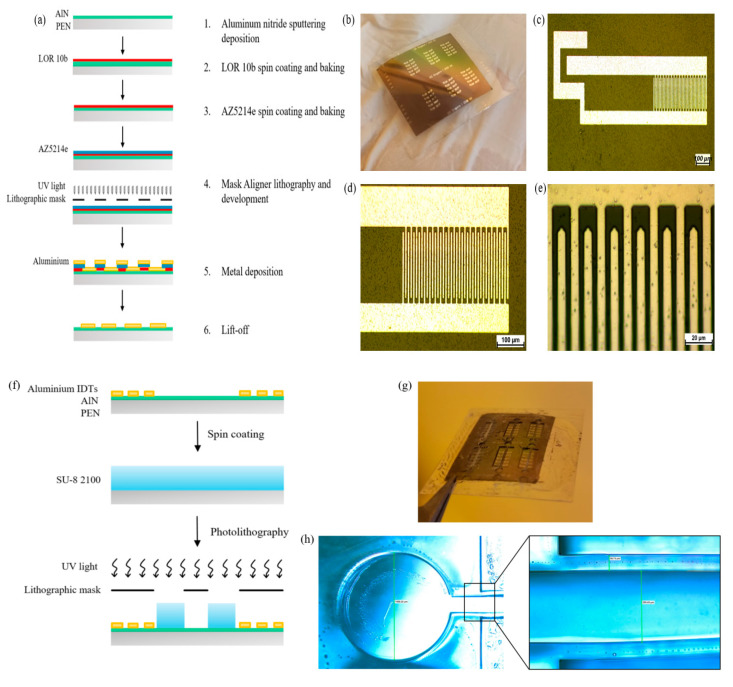
Schematic representation of a bilayer photolithography process (**a**); fabricated SAW device (**b**) and relative electronic microscope images at 10× (**c**), 20× (**d**) and 100× (**e**); schematic representation of SU-8 microchannel photolithography (**f**), fabricated device (**g**) and its microscope images at 4× and 10× (**h**).

The IDTs have been fabricated using bilayer photolithography carried out with Mask Aligner SUSS MA8/BA8 (Garching, Germany). LOR-10B (MicroChem), diluted 1:2 with G thinner (MicroChem, Westborough, MA, USA), was used as non-photosensitive resist followed by an AZ 5214 (MicroChemicals GmbH, Ulm, Germany) photosensitive layer [5]. Samples were washed in acetone, isopropanol and water, respectively, rinsed by N_2_ flow and treated with oxygen plasma (5′/150 W) (RFG300 Deiner, Ebhausen, Germany). Figure 1a shows the steps of the bilayer lithography process. After the photolithography, a 120 nm layer of aluminum was sputtered on the sample through magnetron sputtering deposition (LAB18, Kurt J. Lesker Company, Jefferson Hills, PA, USA). Usually, metal ion-free (MIF) developers are used for the lift-off process [28]. In our case the bilayer resistor was stripped out by Remover PG (MicroChem, Westborough, MA, USA), recommended for LOR-employed lithography. Figure 1b–e shows the microscope images of the fabricated SAW device.

After bilayer photolithography, the sample is subjected to a photolithography process to pattern the microfluidic channel (Figure 1f), using the process described in Table 2 for SU-8 2100 (MicroChem, Westborough, MA, USA), an epoxy-based negative photoresist, which allows us to obtain structures between 100 and 400 μm thick. The microfluidics fabrication employs SU-8 photoresists, which are largely used for making microfluidics channels because of their optical transparency [29], good mechanical and chemical properties once polymerized [30,31] and biocompatibility [32], which are primary features for a biosensing wearable application. Figure 1g shows the whole microfluidic aligned to the SAW device below. The optical images show an inlet diameter of 1500 μm, walls with a width of 50 μm and a microchannel lumen of 250 μm (Figure 1h).

### 2.3. ZnO Nanoparticles’ (NPs) Synthesis and Microchannel Functionalization

Zinc acetate dihydrate Zn (ac_2_) 2H_2_O and sodium hydroxide (NaOH) have been used for the preparation of ZnO NPs. All the chemicals were purchased from Sigma Aldrich (St. Louis, MO, USA) and used without any further purification.

We have grown ZnO NPs following the protocol used by Adam et al. [33] starting from Zn (ac_2_) 2H_2_O 0.1 M and NaOH 0.2 M in deionized water. Then, we put together the two solutions into a becher and stirred at 500 rpm for 2 h under a temperature of 60 °C. Then we applied centrifugation at 4500 rpm for 2 min and the precipitate was washed with deionized water and acetone, respectively. Finally, the sample was dried in an oven at 75 °C for 6 h and ZnO NPs in the form of powder were obtained. 

**Figure 2:**
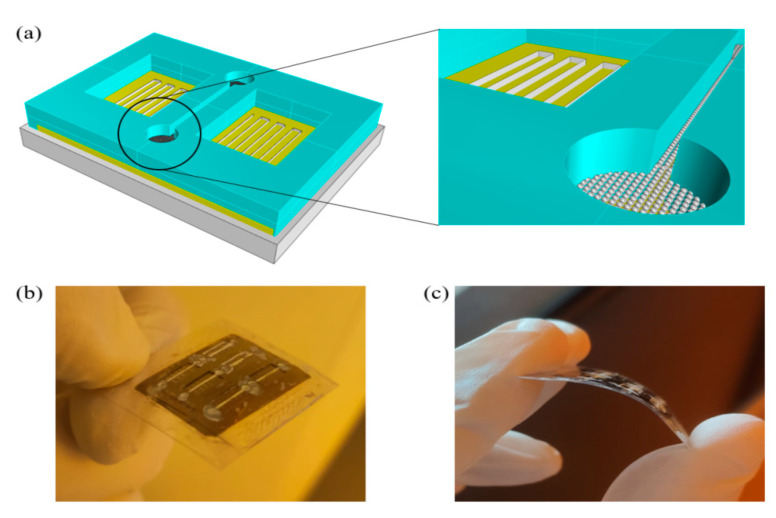
Tridimensional model of finished device (**a**); photo of the fabricated comformable PEN SAW-based device (**b**,**c**).

Finally, microchannels were filled with a ZnO NPs solution of 50 mg/ml in ethanol by drop casting, and internal surfaces were functionalized by drying. NPs were deposited on the ground of the microchannel. The thickness of the deposited layer by drop casting was measured using a Dektak XT Profilometer (Billerica, MA, USA) showing a thickness of about 1.5 μm. This thickness was chosen because pH sensitivity is dependent on NPs layer thickness. Oh et al. have demonstrated a better linear response for a thickness around 1 μm [2]. After these steps, the finished device (Figure 2) was tested.

### 2.4. Buffer Solutions Preparation

All chemicals used to prepare buffer solutions for pH tests were purchased from Sigma Aldrich (St. Louis, MO, USA). In particular, HCl/KCl buffer has been used to prepare pH = 2 solution, sodium citrate dihydrate/citric acid buffers have been used for solutions from pH = 3 to pH = 6, and MilliQ water was used for pH = 7. All solutions’ pH values have been adjusted to the final desired pH using HCl or NaOH. A range of pH between 2 and 7 was chosen for this work in order to test the physiological value of pH in sweat.

## 3. Results and Discussion

### 3.1. Mechanical Properties

**Figure 3:**
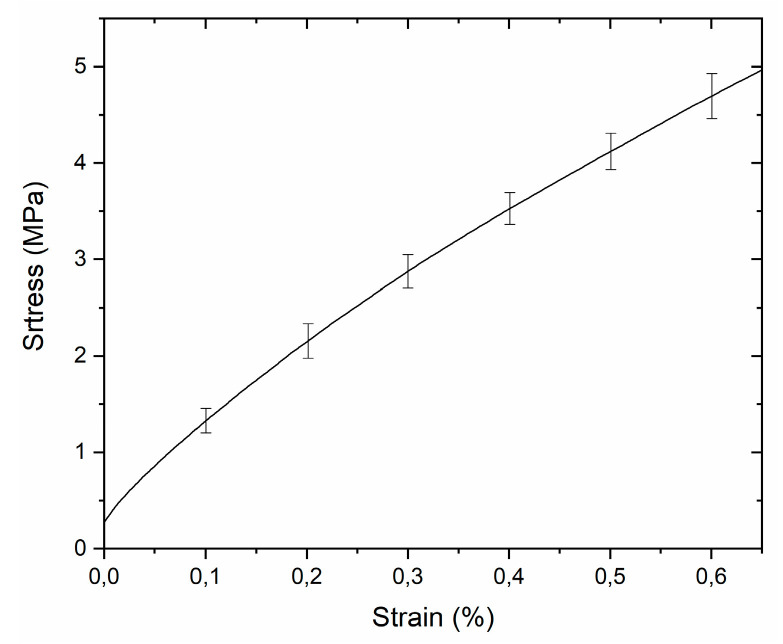
Resulting stress/strain curve in the linear-elastic range of the tensile strength of a PEN/SU-8 hybrid device.

Mechanical characterization is fundamental to verify the conformability of a new hybrid material to human skin. Such devices should show the typical Young’s modulus of the skin, which can be obtained through flexible substrates [34]. For this reason, the mechanical properties of the new PEN/SU-8 hybrid device (t_PEN_ ~ 125 μm, t_SU-8_ ~ 300 μm) were assessed with a dynamic mechanical analyzer (DMA Q800, TA Instruments, USA). In particular, tensile strain was investigated (Figure 3).

Strip samples with dimensions of 40 × 10 mm^2^ were used for tensile tests and a ramp force of 1 N/min to 18 N at 25 °C was used. In the linear-elastic region, Hooke’s law can be applied to describe sample responses. The resulting Young modulus of the hybrid device was 0.92 ± 0.04 GPa, comparable with the value of other flexible devices employed in skin-placed flexible biosensors [34].

### 3.2. Chemical–Physical Properties of Microfluidic Functionalization

**Figure 4:**
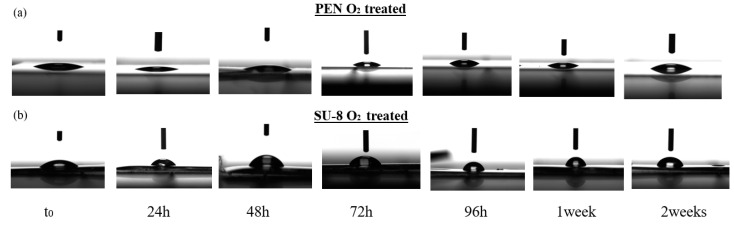

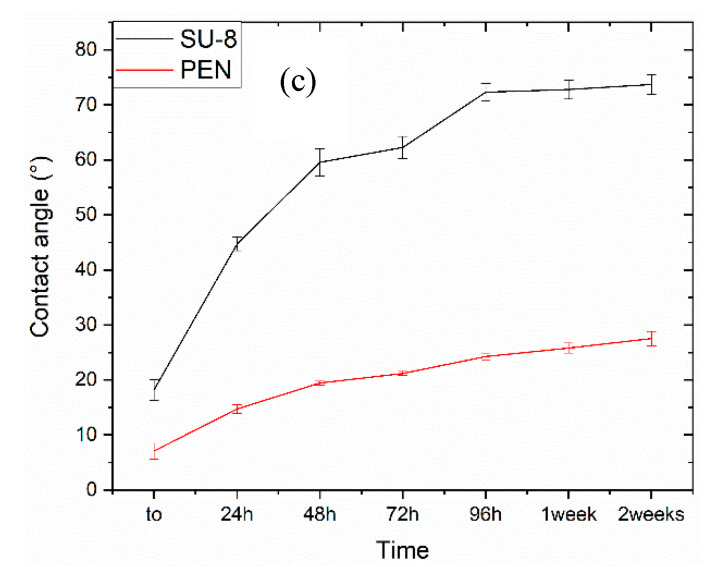
Images of PEN (**a**) and SU-8 (**b**) aging contact angle test and relative measurements at t_0_, 24 h, 48 h, 72 h, 96 h, one and two weeks (**c**).

The hydrophilicity of the channel is a key parameter in microfluidic SAW devices. The microfluidic channel must be highly hydrophilic to allow a water solution to pass through by use of capillary force [35]. For this reason, the contact angle measurements (undertaken with a Contact Angle System Oca 15 Pro (DataPhysics, Filderstadt, Germany) have been carried out using deionized water. PEN and SU-8 show contact angles of 80.92 ± 2.73° and 85.10 ± 2.23°, respectively; these values show a weak hydrophilic character, unsuitable for microfluidics. In order to reduce the contact angle, an O_2_ plasma treatment has been performed on both substrates using an Oxygen Plasma Asher RFG 13.56/300 (Diener electronic, Ebhausen, Germany). An oxygen flow of 25 sccm at a pressure of approximately 0.6 mbar and at a power of 230 Watt was used for 5 min. We performed contact angle goniometry to characterize the wetting behavior of the plasma-activated SU-8 and PEN surfaces, and the measurements at t_0_ show a huge decrease in the contact angle, which was 7.06 ± 1.41° for PEN and 18.19 ± 1.88 for SU-8 (Figure 4a,b). An aging test was conducted to analyze the efficacy through time of the treatment [36,37]. Aging measurements on PEN show a contact angle of 27.52 ± 1.32° after two weeks, with a negligible variation of about 20° since t_0_. On the other hand, SU-8 shows an angle of 73.71 ± 1.82° after two weeks, with a stabilization of the value after 96 h (Figure 4c); hence, even with SU-8 partially losing its hydrophilic character, it still has enough to allow water to go across it. 

### 3.3. ZnO Nanoparticles (NPs) Characterization

**Figure 5:**
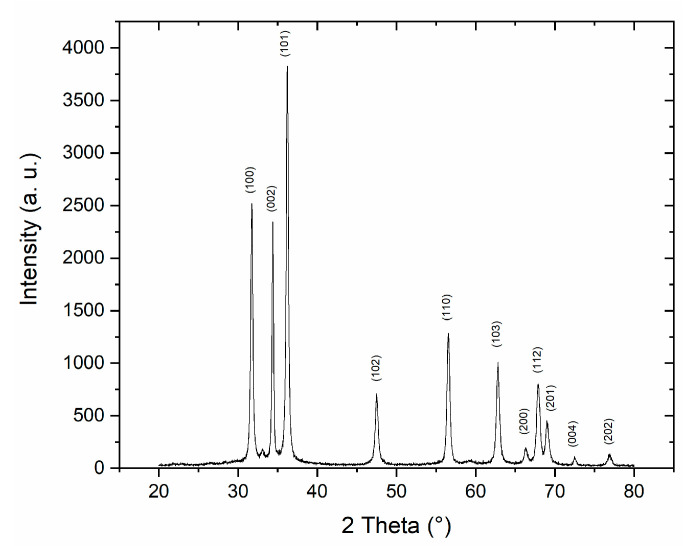
XRD spectrum of ZnO NPs.

The ZnO NPs have been characterized using X-ray diffraction (XR) to evaluate the crystallographic quality of the sample using a Rigaku D-Max/Ultima (Tokyo, Japan) working at 40 kV with a Cu Kα radiation. In the XRD pattern (Figure 5), the resulting peaks correspond to the pure hexagonal wurtzite phase of ZnO [33]. These results allowed us to see that no other ZnO phases or impurities are present in the sample. 

The crystalline size was calculated using the Scherrer equation:

(6) $$D=\frac{k\lambda }{\beta \;\cos\;\theta }$$

where D is the crystalline size of ZnO, λ is the X-ray wavelength (0.15406 nm), θ is the Bragg diffraction angle, β is the full-width at the half maximum (FWHM) of the diffraction peak corresponding to plane (101) and k is Scherrer constant (0.9). The resulting average crystalline size of ZnO, calculated with this formula, is 25 nm.

**Figure 6:**
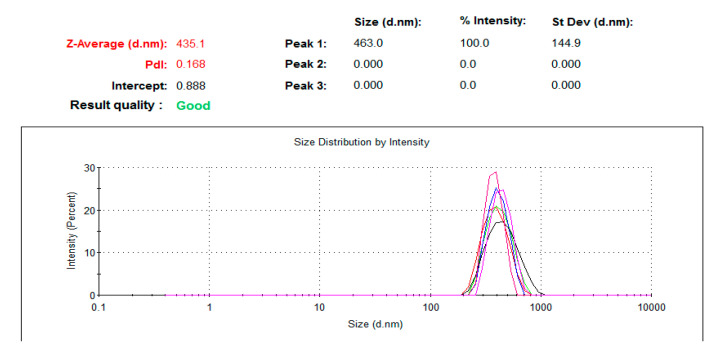
DLS analysis of ZnO NPs.

NPs dimensions, dependent on the speed of the addition of zinc acetate to NaOH solution during the synthesis [38], was evaluated using a dynamic light scattering (DLS) test through a Zetasizer Nano (ZS) (Malvern Panalytical, Malvern, UK)). The concentration of the samples was 1 mg/mL in a deionized water solution, and before the test they were sonicated for 90 min. The results show NPs of 435.1 ± 26.4 nm (Figure 6). Only one peak in the distribution was observed, demonstrating a uniform distribution of the dimensions.

**Figure 7:**
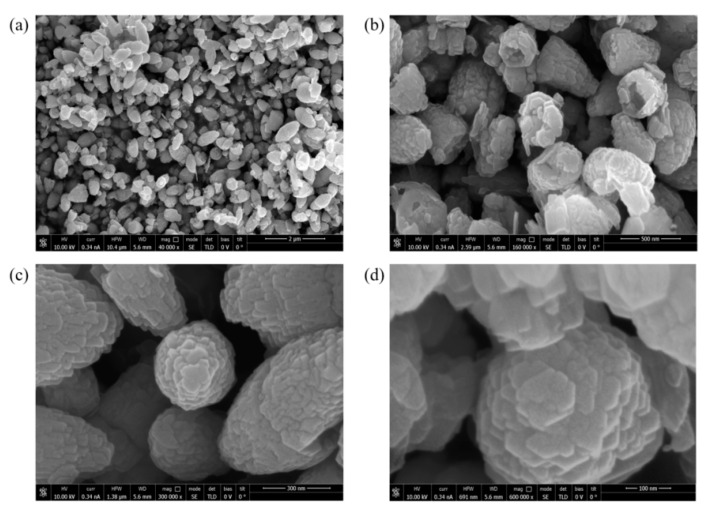
SEM images of the ZnO NPs at different magnifications; 40,000× (**a**), 160,000× (**b**), 300,000× (**c**) and 600,000× (**d**).

The morphology of the synthetized NPs has been evaluated by SEM analysis using a FEI Helios Nanolab 600i Dual Beam FIB/SEM system (Waltham, MA, USA). Before the SEM analysis, the samples were covered with a thin, 5 nm layer of gold with a sputter coater (Quorum, Q150 R S, Lewes, UK)) in order to improve the quality of the images. Via SEM, the shapes of the ZnO NPs have been characterized (Figure 7a,b). The NPs were uniform and showed a crystalline average size of ≈25 nm as calculated by the Scherrer equation. Furthermore, the dimensions of the ZnO NPs are coherent with the results of the DLS analysis, in which was found a dimension of about 430 nm. Finally, high magnifications allowed us to see the hexagonal wurtzite shape (Figure 7c,d).

### 3.4. Electroacoustic Response of the SAW Device

**Figure 8:**
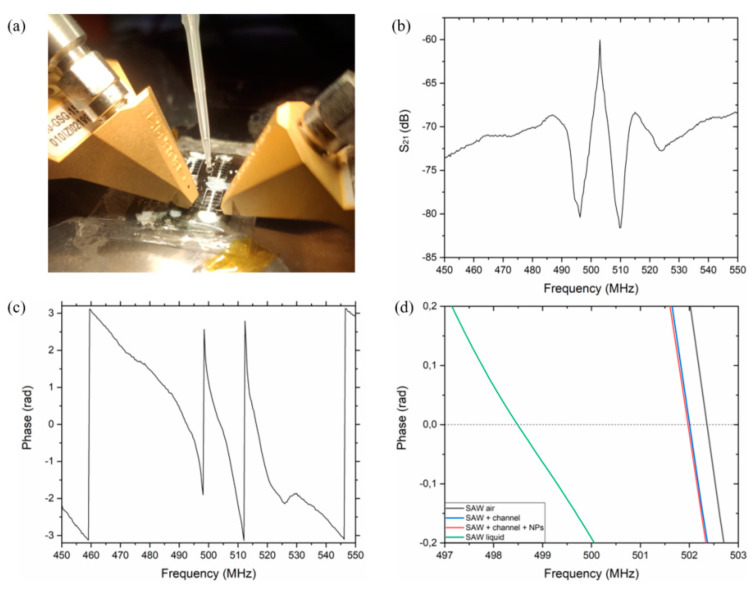
Experimental setup, used to study the electroacoustic response of the SAW devices (**a**), transfer function amplitude S_21_ of the PEN device in air (**b**) and its phase response in air (**c**); changes in phase response of SAW device in air, after microchannel fabrication, after NPs deposition and the microchannel was filled with liquid (**d**).

The devices were connected to the VNA (Agilent 8753ES, Santa Clara, CA, USA) with a coplanar |Z| Probe (Electron Mec) with a 150 µm pitch and they were tested by measuring the transmission amplitude signal S21, obtained by a Vector Network Analyzer (Tektronix, TTR503A). The setup in Figure 8a was used to test the device at different pH values. All the devices have been tested in a controlled environment at 25 °C and 30–40% of humidity. PEN devices show a clear resonance frequency corresponding to the propagation of Lamb SAW around 500 MHz. A transmission signal of about 15 dB is observed for these waves and a good phase response is clearly visible (Figure 8b,c). In this work all the devices were tested before the fabrication of the microchannel, after its fabrication and after the functionalization with NPs, to record the reference frequency. A significant frequency resonance decrease of about 375 kHz was observed due to the SU-8 microchannel, because of its weight, but no significant decrease was observed after the functionalization with NPs due to their low weight. A significant frequency shift of ≈4 MHz was observed when the first solution (pH = 7) was added (Figure 8d).

**Figure 9:**
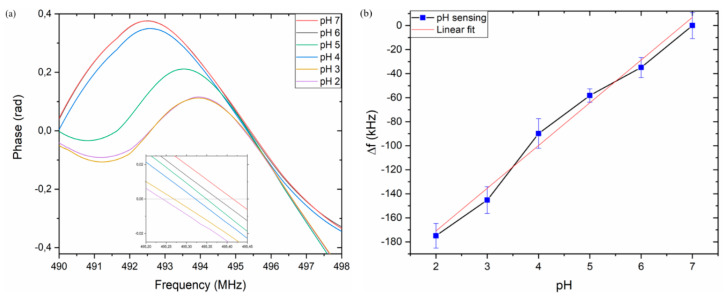
Phase change at different pH solutions, inset in figure shows a zoom of phase response (**a**); resonance frequency shift in response to different pH values (**b**).

Six solutions from pH = 7 to pH = 2, with a step of one unit, have been used to evaluate the electroacoustic response in the L-SAW resonance. The presence of a liquid medium induces a shift of about 3.7 MHz to the frequency of the device in air (Figure 8d). The resonance center frequency obtained with the pH = 7 solution has been used as a reference frequency, f0, in the experiment. In Figure 9a, the phase responses for PEN devices at different pH values are shown. The inset in Figure 9a clearly shows the center frequency shift for the different pH values. Figure 9b reports the resonance shift (Δf = f − f_0_), where f_0_ represents the center frequency at pH = 7 and f the center frequency of the other pH values. As expected, the center frequency of the sensor response decreases for lower pH levels due to the increase in ZnO NPs conductivity. The same device has been used to perform measurements at different pH values, after washing and drying the microchannel after each measurement, demonstrating that the shifts are reversible, and the device is reusable. In fact, according to Oh et al., no dissolution of ZnO NPs has been observed after exposure to a strong acid [2].

A sensitivity of ≈30 kHz/pH has been observed; a linear response is obtained with a regression slope value with an R^2^ = 0.983 and a total shift of ≈173 kHz in the pH dynamic range between 2 and 7.

## 4. Conclusions

A novel SU-8-based microfluidic, reusable, PEN/AlN SAW device based on ZnO NPs for pH sensing has been designed, fabricated, characterized and tested. The method consists of patterning a ≈300 μm thick microfluidic channel between the two IDTs on a SAW device; then, the microchannel functionalization with ZnO NPs makes it sensitive to pH, due to the ZnO NPs’ changing conductivity with pH, and the SAW Lamb resonance frequency of about 500 MHz. The microfluidic channel has shown a Young’s modulus of 0.92 ± 0.04 GPa, compliant with a wearable device, and a water contact angle of 27.52 ± 1.32° and 73.71 ± 1.82° for the PEN and SU-8 surfaces, respectively, demonstrating their hydrophilic character. The electroacoustic response has shown a sensitivity of ≈30 kHz/pH and a total shift of ≈173 kHz. To our best knowledge, this is the first time a microfluidic channel has been directly fabricated on a SAW flexible device for sensing applications. A specific functionalization of the microchannel could make it sensitive to specific biological parameters, such as cells mass loading, bacteria, viruses, and small molecules masses, paving the way to the miniaturization of the electroacoustic-based detection of biological samples in liquid.

## Figures and Tables

**Table 1:** Design parameters of the IDT electrode of SAW fabricated devices.

**N° Fingers**	40 Pairs
**Wavelenght (λ)**	20 μm
**Metallization Ratio**	0.5
**Acoustic Aperture**	10 λ
**Distance between IDTs**	20 λ

**Table 2:** Microfabrication process of ~300 μm thick microfluidic channel flow process.

**Sample Cleaning**	Acetone/Isopropanol/Water
**Drying**	N_2_ flow
**Spin Coating of SU-8 2100**	1000 rpm for 60″
**Soft Bake**	5′ at 65 °C (reduce stress on the film) 95′ at 95 °C
**Exposure**	360 mJ/cm²
**Post Exposure Bake (PEB)**	10′/65 °C
**Development**	8–9′ in SU-8 developer
**Rinse and Dry**	Water and N_2_ flow
